# Populations of Latvia and Lithuania in the context of some Indo-European and non-Indo-European speaking populations of Europe and India: insights from genetic structure analysis

**DOI:** 10.3389/fgene.2024.1493270

**Published:** 2024-11-20

**Authors:** Gintė Daniūtė, Laura Pranckėnienė, Jurgis Pakerys, Jānis Kloviņš, Vaidutis Kučinskas, Alina Urnikytė

**Affiliations:** ^1^ Department of Human and Medical Genetics, Biomedical Science Institute, Faculty of Medicine, Vilnius University, Vilnius, Lithuania; ^2^ Faculty of Medicine Population Genomics Laboratory, Translational health research Institute, Faculty of Medicine, Vilnius University, Vilnius, Lithuania; ^3^ Department of Baltic Studies, Institute for the Languages and Cultures of the Baltic, Vilnius University, Vilnius, Lithuania; ^4^ Latvian Biomedical Research and Study Centre, Riga, Latvia

**Keywords:** Lithuanian population, Latvian population, Indian population, genetics, population structure

## Abstract

The aim of this study was to investigate the relationship among Lithuanian, Latvian, Indian, and some other populations through a genome-wide data analysis of single nucleotide polymorphisms (SNPs). Limited data of Baltic populations were mostly compared with geographically closer modern and ancient populations in the past, but no previous investigation has explored their genetic relationships with distant populations, like the ones of India, in detail. To address this, we collected and merged genome-wide SNP data from diverse publicly available sources to create a comprehensive dataset with a substantial sample size especially from Lithuanians and Latvians. Principal component analysis (PCA) and admixture analysis methods were employed to assess the genetic structure and relationship among the populations under investigation. Additionally, we estimated an effective population size (*Ne*) and divergence time to shed light on potential past events between the Baltic and Indian populations. To gain a broader perspective, we also incorporated ancient and modern populations from different continents into our analyses. Our findings revealed that the Balts, unsurprisingly, have a closer genetic affinity with individuals from Indian population who speak Indo-European languages, compared to other Indian linguistic groups (such as speakers of Dravidian, Austroasiatic, and Sino-Tibetan languages). However, when compared to other populations from the European continent, which also speak Indo-European and some Uralic languages, the Balts did not exhibit a stronger resemblance to Indo-European-speaking Indians. In conclusion, this study provides an overview of the genetic relationship and structure of the populations investigated, along with insights into their divergence times.

## Introduction

The origins, spread, and timing of the Indo-European language family, which gave rise to the main languages of the Baltic countries and to many languages of India, have been subjects of debate. One of the main proposals, the “Steppe” hypothesis, suggests that the spread of Indo-Europeans occurred from the Pontic Steppe ca. 6,500 years BP (Gimbutas 1970; Mallory, 1989; Anthony, 2007) The alternative “Anatolian” hypothesis proposes that the expansion originated from Anatolia region and occurred much earlier, around 9,500–8,500 years BP (Renfrew, 1987; Bellwood, 2005). In addition to that, one of the recent studies proposed a hybrid model: here, the primary homeland is seen south of the Caucasus (as in the “Anatolian” hypothesis) while the later secondary homeland is located in the steppe region (in line with the “Steppe” hypothesis) (Heggarty et al., 2023). As for development of the languages that are of primary importance for our study, it is widely accepted that the Baltic languages grew out of the Balto-Slavic branch of the Proto-Indo-European, while the Indo-European languages of India are surely known to have evolved from the Indo-Iranian branch. The common linguistic features of the Balto-Slavic and the Indo-Iranian languages were inherited from the stages of diversification of the Proto-Indo-European and the two branches do not seem to have shared an exclusive common ancestor (Pronk, 2022; Kümmel, 2022). In some studies, however, a potential ancestor of the Indo-Iranian and the Balto-Slavic protolanguages is proposed and is referred to as Indo-(Iranian-Balto-)Slavic node (Ringe et al., 2002; Nakhleh et al., 2005; Olander, 2019); see the latest critical evaluation of this proposal in (Heggarty et al., 2023). Despite the well-researched linguistic relationship, there have been no detailed studies investigating the genetic relationship between geographically distant Baltic and Indian populations and the present study aims to fill this gap. The results of this study could potentially lead to a wider-scale examination of the genetic relationship between the Balto-Slavic and the Indo-Iranian speaking populations.

The territory of the present-day Baltic countries, Lithuania and Latvia, was first inhabited during the Final Paleolithic ca. 11,000–10000 years BP by the hunter-gatherers (HG) who were migrating as the climate was warming up after the last glaciation (Rimantienė, 1996; Zagorska 2001; Ostrauskas, 2008; Jochim, 2011). HG populations were dominant in the area up until ca. 5,000–4500 BP and left a lasting impact on the genetic profile of inhabitants of Lithuania and Latvia which are known to carry the largest share of west European HG ancestry in Europe (Urnikyte et al., 2019; Reščenko et al., 2023). An event of major importance for further formation of the Baltic populations was the arrival of Indo-European speaking populations which ca. 5,000–4,500 years BP reached Europe through migration waves from the Pontic Steppe (Allentoft et al., 2015; Haak et al., 2015). The migrant populations merged with the local ones and subsequently gave rise to the Baltic-speaking populations via the intermediate stage of Balto-Slavic unity (Pronk, 2022). Throughout the history, the Baltic populations were influenced by neighboring non-Indo-European speaking (Finnic) as well as Indo-European-speaking (Germanic, Slavic) populations. Despite certain admixture, the contemporary Lithuanians and Latvians are considered relatively homogeneous populations, with the genetic diversity of Baltic tribes diminishing over the past millennium due to the geographical isolation of these lands (Kasperavičiūtė et al., 2004; Pliss et al., 2015; Ruzgaitė et al., 2015; Urnikyte et al., 2019). In contrast, India is known for its high genetic, cultural and linguistic diversity (Majumder, 1998). Indo-European migrations have also played a significant role in the formation of the Indian population (Diamond and Bellwood, 2003; Pugach and Stoneking, 2015). Indian populations are divided into tribal and caste groups, with castes comprising most of the population (Basu et al., 2003). There are approximately 400 tribal groups in India, speaking hundreds of languages, which belong to the four major language families: the Indo-European (in northern India), the Dravidian (in southern India), the Sino-Tibetan (in northeastern India), and the Austroasiatic (in fragmented areas of eastern and central India) (Majumder and Basu, 2015).

Recent studies have shown that Lithuanian and Latvian populations are genetically close to their neighbors: Estonians (speaking a Finnic language of the Uralic language family), and Belarusians and Poles (speaking East and West Slavic languages of the Indo-European family respectively) (Urnikyte et al., 2021). Most Indian groups have inherited of their ancestry from the Ancestral North Indians (ANI) related to Middle Easterners, Caucasians, Central Asians, and Europeans, and Ancestral South Indians (ASI) who are distantly related to West Eurasians (Reich et al., 2009; Metspalu et al., 2011; Moorjani et al., 2013). Furthermore, individuals from higher castes within Indian population have been found to be genetically more closely related to Europeans than Asians (Heggarty et al., 2023; Bamshad et al., 2001; Thanseem et al., 2006; Bose et al., 2021).

## Materials and methods

### Samples

#### The baltic context

We made use of samples from 416 unrelated adult participants from two major ethnolinguistic groups (Aukštaitija (N = 218) and Žemaitija (N = 198)) in Lithuania. All participants indicated a minimum of three generations of Lithuanian ethnicity. The data used were from LITGEN project, already used in previous paper (Urnikyte et al., 2019). To get a diverse dataset of the Baltic populations, we merged Lithuanian samples with Latvian population samples. The 287 Latvian sample data is published by the Latvian Biomedical Research and Study center (LVBMC) (Reščenko et al., 2023). Participants reflect all the regions and the major ethnic groups of Latvia (Courland (N = 61), Semigallia (N = 24), Vidzeme (N = 147), Latgale (N = 55)) (see Supplementary Table S1.1). After merging we ended up with a total of 703 samples, and 153,244 SNPs. All the study participants signed written informed consent in compliance with the Declaration of Helsinki.

### The Indian context

The genome wide SNP data of 456 Indians was obtained from the following publicly available sources: 102 samples from Telugu (South Asian Ancestry) ethnic group living in United Kingdom and 103 samples from Gujarati (South Asian Ancestry) ethnic group currently residing in Houston, Texas (The 1000 Genomes Project Consortium et al., 2015); 140 samples from 29 ethnic groups from Metspalu et al., 2011 study (Metspalu et al., 2011); 22 samples from 6 ethnic groups from Tätte et al. (2019) study (Tätte et al., 2019); 44 samples from 3 ethnic groups from Pathak et al. (2018) study (Pathak et al., 2018); 19 samples from Parsi ethnoreligious group from Chaubey et al. (2017) study (Chaubey et al., 2017); 26 samples from 10 ethnic groups from Chaubey et al. (2010) study (Supplementary Table S1.2). In total, we generated a combined dataset of 343,295 SNPs from a total of 1,159 subjects from Lithuania (N = 416), Latvia (N = 287), and India (N = 456).

For further analysis, to see a broader view in the context of other world populations, the generated pooled dataset was merged with 36 samples from Estonian (Tambets et al., 2018) and with 17 populations from 1000 Genomes project (The 1000 Genomes Project Consortium et al., 2015) (Supplementary Table S1.3). In total this dataset consisted of 2,883 individuals and 343,295 SNP.

Lithuanian, Latvian, and Indian dataset was also merged with a dataset of ancient and recent samples from Eurasia. Besides Lithuanians, Latvians and Indians, this dataset consisted of 406 ancient and recent samples from Lazaridis et al. (2016) study (Lazaridis et al., 2016), 17 recent samples from Behar et al. (2013) study (Behar et al., 2013), 54 ancient samples from Mathieson et al., (2018) study (Mathieson et al., 2018) and 11 ancient samples from Mittnik et al. (2018) study (Mittnik et al., 2018) (Supplementary Table S1.4). In total this dataset consisted of 1,647 individuals and 13,522 SNPs.

Datasets were merged and processed using *plink* v1.90, *VCFtools* v.01.16 and *bcftools* v.1.14 programs (Purcell et al., 2007; Danecek et al., 2011; Danecek et al., 2021).

## Methods

### Principal component analysis, admixture, and outgroup f3-statistics

Principal component analysis (PCA) was performed with SmartPCA package from EIGENSOFT v7.2.1 (Patterson et al., 2006) on the pruned SNPs. SNPs in linkage disequilibrium were removed with the indep-pairwise option of PLINK (v1.07) using a window size of 50 SNPs, a step size of 5, and a r2 threshold of 0.5.

Inbreeding coefficients (F) among individuals from Lithuanian, Latvian and Indian populations was measured using plink v1.90 program (Purcell et al., 2007). Negative F values were changed to zeros as they probably represent sampling errors. Kinship was measured using KING v.2.2.5 (Manichaikul et al., 2010). PCA outliers, individuals having F than that expected for second cousin mating offspring (F ≥ 0.0156) and second-degree relatives (kinship coefficient >0.0884) were omitted from all further analyses (in total 17 samples), except for Indian samples (Supplementary Table S2.1). PCA results were plotted using R v4.3.1 software ggplot2 package (Wickham, 2016).

Assessment of each individual genetic ancestry was performed with ADMIXTURE v1.3.0 (Alexander et al., 2009). In analysis we included both present-day and ancient samples with K from 2 to 12 with 10 iterations. The results were plotted using the PONG tool (Behr et al., 2016).

Outgroup f3-statistics were computed using qp3Pop program from ADMIXTOOLS V4.1 (Patterson et al., 2012). Mbuti was considered as outgroup in the analysis and calculated the shared drift between each putative ancient group and all the modern groups in the dataset in the form (Mbuti; Ancient, Modern).

### Effective population size and divergence time

The long-term *Ne* and divergence time for the populations under study were estimated using R Package NeON (Neon, 2015) based on the genetic distance between SNPs. For the analysis we used binary PLINK files and updated genetic map information of the markers to calculate the *Ne* over time. First, the squared correlation coefficient of linkage disequilibrium (*r*2*LD*) within predefined recombination distance categories between markers is estimated. We used a function that generates 250 overlapping recombination distance categories with a step of 0.001 centiMorgan (cM) from 0.005 to 0.25. Then, *Ne* with a confidence interval 95% for each recombination distance category applying the formula Ne ≈ 1/(4c) * [(1/r2)-2] is calculated (c is the distance between markers in Morgan). The long-term *Ne* was estimated as the harmonic mean of the effective population size along the generations in the past for each population (Wright, 1931). Knowing the values of *Ne* and having the matrix of the calculated pairwise *F*_*ST*_ values with 4P software (Benazzo et al., 2015), we could estimate the time of divergence between populations using the *Tdverg* function of the NeON R package. Divergence time between pairs of study populations in generations was estimated as follows: *T = ln(1 − F*_*ST*_ *)/ln(1–1/2Ne*), where T represents divergence time. A generation is assumed to be 25 years long. The evolutionary history based on estimated divergence times was inferred using the UPGMA method implemented in MEGA X software v.10.2.5 (Kumar et al., 2018).

## Results

### Principal component analysis results on Baltic and Indian populations

Principal component analysis (PCA) was performed on genome wide 343,295 SNP data of 1,142 individuals, which were distinguished by the country of origin (Lithuania, Latvia, and India). We revealed 2 Latvian and 5 Indian populations outliers (Supplementary Figure S2.1), which were subsequently removed from further analyses.

The Indian population was classified based on the families of languages they mostly speak (Indo-European, Dravidian, Austroasiatic, and Sino-Tibetan). This classification was chosen because it provides information about geographical location of the samples (Supplementary Table S1.2). Samples from the Lithuanian and Latvian populations were distinguished solely by their country of origin, as they exhibited a high level of homogeneity and formed a single cluster. This approach was adopted in order to accurately represent the diversity within the Indian population and to ensure that meaningful conclusions can be drawn from the analysis (Supplementary Figure S2.1).

**FIGURE 1:**
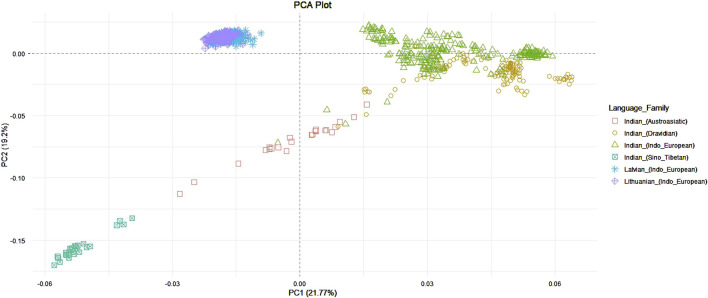
PCA among Lithuanian, Latvian, and Indian language families.

The first two principal components explained 45,49% of genetic variance among populations and showed that individuals from Lithuanian and Latvian populations form one cluster, while individuals from the Indian population form a distinct group. Also, as quite expected, the Indian population had a much bigger data dispersion in the plot when compared to the Balts. This shows a higher genetic diversity of the Indian population due to a complex interplay of historical migrations, long-term population structure, and social factors like the caste system and linguistic diversity. The samples of Indian speakers of Indo-European languages showed a closer allocation to the Balts compared to other linguistic groups of Indians. The most distant from the Balts are samples which belong to the speakers of Sino-Tibetan languages (Figure 1).

### Principal component analysis results on baltic, indian and other world populations

**FIGURE 2:**
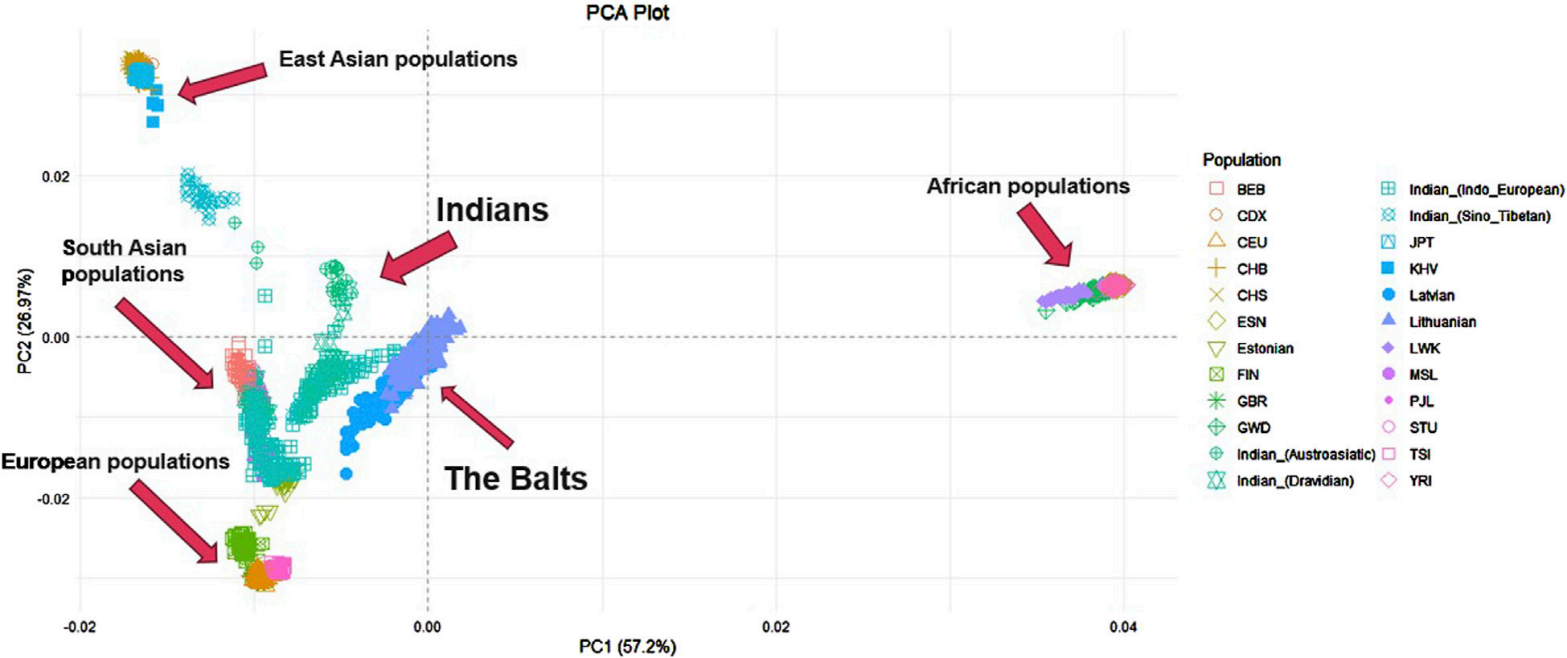
PCA of Lithuanian, Latvian, Indian and other world populations. YRI - Yoruba in Ibadan, Nigeria, LWK - Luhya in Webuye, Kenya, GWD - Gambian in Western Divisions in the Gambia, MSL - Mende in Sierra Leone, ESN - Esan in Nigeria, CEU - Utah Residents with Northern and Western European Ancestry, TSI - Toscani in Italia, FIN - Finnish in Finland, GBR - British in England and Scotland, CHB - Han Chinese in Bejing, China, JPT - Japanese in Tokyo, Japan, CHS - Southern Han Chinese, China, CDX - Chinese Dai in Xishuangbanna, China, KHV - Kinh in Ho Chi Minh City, Vietnam, PJL - Punjabi from Lahore, Pakistan, BEB - Bengali from Bangladesh, STU - Sri Lankan Tamil from the UK.

Further PCA was performed on the Lithuanian, Latvian, Indian dataset merged with other world populations from 1000 Genomes project. In total it included 2,859 individuals and 343,295 SNP. Individuals from the Indian population were again distinguished based on the family of their main language to get a more accurate view and see which linguistic group from this large diverse population has a greater resemblance to the Balts. The first principal component explained 57,2% of genetic variance among populations, the second principal component – 26,97% (Figure 2). The first principal component distinguishes ESN, GWD, LWK, YRI and MSL populations (Africa) from other analyzed populations. The second principal component distinguishes KHV, JPT, CHB, CHS populations (East Asia) from populations of Europe and South Asia. Lithuania and Latvia together form a distinct cluster but are closer to the Indian populations, GIH, PJL, STU, BEB (South Asia) and Estonians (Europe). Close to these clusters another group is found of TSI, FIN, CEU and GBR populations (Europe). The Indian population has the biggest data dispersion in the plot when compared to other populations. Indo-European and Dravidian speaking individuals cluster closer to the Baltic populations than Sino-Tibetan or Austroasiatic speaking individuals.

### Principal component analysis results on baltic, indian and ancient and modern populations from eurasia

**FIGURE 3:**
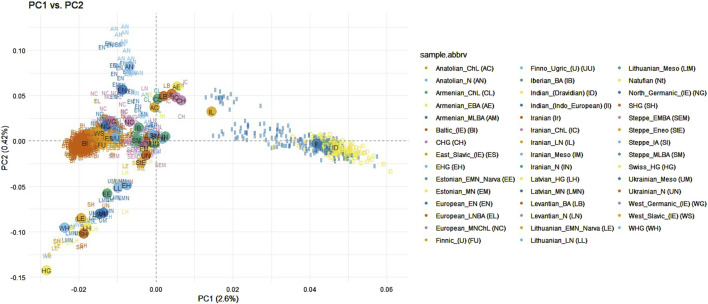
PCA of Indian (Indo-European and Dravidian speaking), ancient and modern Eurasian populations when modern populations were distinguished by language subfamilies. Abbreviations of ancient populations are explained in Supplementary section table 1.4.

Principal component analysis was conducted on the dataset by merging the samples from the Baltic and Indian populations with those from other ancient and modern populations across the Eurasian continent (as shown in Supplementary Figure S1.4). The purpose of this analysis was to determine whether ancient populations, particularly those from Anatolian and Steppe regions, exhibit a closer genetic relationship to Indo-European speaking populations. This investigation also aimed to evaluate the hypotheses of the homeland of the Indo-European languages discussed in the Introduction. Some modern populations from the European continent were included in the analysis with the objective to investigate whether the speakers of the Baltic languages share a stronger genetic connection with the Indian population compared to other neighboring European populations. This dataset consisted of 1,628 samples and 13,522 SNPs. Indian ethnolinguistic groups which were assigned to Austroasiatic or Sino-Tibetan languages families were removed from analysis since they have been shown as clearly distant from the Baltic populations in the previous analysis (Figure 1). After filtering, the dataset consisted of 1,569 samples and 10,208 SNPs. First principal component analysis revealed 1 Levantian Neolithic and 2 Iranian Neolithic outliers which were removed from the further analysis (Supplementary Figure S2.4). Ancient samples were assigned to populations as in Supplementary section table 1.4., and individuals from modern populations were distinguished based on the language branches and subbranches, the Indo-European family is represented by Baltic (Lithuanian and Latvian populations), East Slavic (Belarusian, Russian, Ukrainian populations), West Slavic (Polish population), North Germanic (Icelandic, Norwegian populations), West Germanic (English and Scottish populations), the Uralic family is represented by Finnic (Estonian and Finnish populations), and Mordvin (Mordovian population). The first principal component explained 2.6% of genetic variance and distinguished the Indian populations from the analyzed modern Eurasian populations. However, Indo-European speaking Indians clustered closer to the Eurasian populations compared to the Dravidian speaking Indians. Balts display a tight cluster partly overlapping the West Slavic, and North Germanic groups with close proximity towards one of the extremes of the Finnic population clusters (Figure 3). Compared to ancient samples, modern Balts and Finns positioned as the closest present-day European populations to ancient hunter-gatherer groups (Latvian HG, Western HG, Eastern HG, Scandinavian HG), neolithic ancient samples from Lithuania, Estonia and Latvia, also Ukrainian Mesolithic and neolithic samples, and showed high proximity to the Steppe and late Neolithic Bronze Age European samples (Figure 3). The Balts did not show a closer genetic relationship with Indians compared to other Eurasian populations (Figure 3).

### Admixture analysis

The dataset of ancient and modern samples from Eurasian continent was used, with previously mentioned outliers and Indian populations speaking Austroasiatic or Sino-Tibetan languages removed. This dataset consisted of 1,569 samples and 10,208 SNPs. We included 17 modern populations (Lithuanian, Latvian, Belarusian, Ukrainian, Russian, Polish, English, Scottish, Orcadian, Icelandic, Norwegian, Iranian, Indian (Indo-European), Indian (Dravidian), Mordovian, Estonian, Finnish (Uralic)) and a group of 34 ancient populations. The best CV value of admixture analysis was determined when populations were distributed into 4 clusters (Supplementary Table S2.2).

Admixture analysis revealed that the Baltic population is characterized by three main genetic components reflecting Hunter Gatherers ancestry (in green), other components found in the European Neolithic/Anatolian Neolithic (in purple) and in the Iranian, Lithuanian Mesolithic, Lithuanian EMN Narva (red). Indians are also characterized by three genetic components–blue found maximized in Lithuanian and Estonian EMN Narva, Lithuanian Late Neolithic, and Estonian Middle Neolithic; red found in the Iranian, and purple which is observed only in one part of Indo-European speaking Indians and found in the European Neolithic/Anatolian Neolithic samples. All modern European populations are mainly characterized by three color compounds as well as the Balts. Some of them, however, have a small part of purple color (West Slavs). Out of modern European populations, the Polish population has the biggest part of the blue color component, which is characteristic to Indians. From the ancient populations, Lithuanian Early-Middle Neolithic Narva samples have the biggest amount of blue color (Supplementary Figures S3, S4). The full admixture analysis is provided in Supplementary Figure S2.8, S2.9.

**FIGURE 4:**
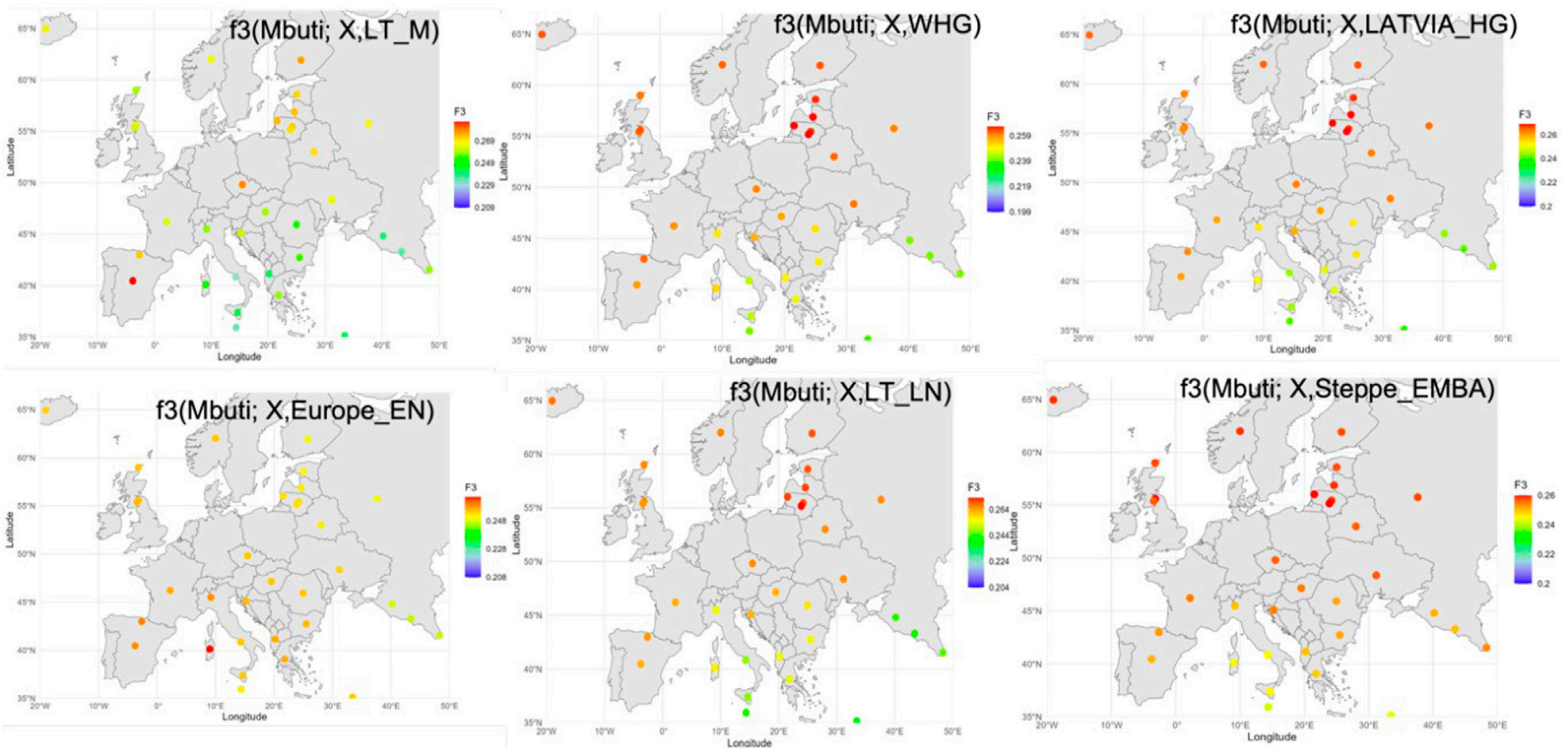
Geographical distribution of outgroup f3-statistics showing shared genetic drift top values between antient and modern samples analysed. Each analysed modern population is displayed in its corresponding location on the map. The darker the red color, the higher the pairwise f3 statistics between modern population and the ancient population group, representing higher allele sharing.

Subsequently we used outgroup-f3 statistics (Patterson et al., 2012) to measure shared ancestry between Baltic and ancient populations from different geographic regions. Among the modern populations of the Lazaridis et al., (2016) dataset (Lazaridis et al., 2016), Balts showed the highest f3 values when testing Western HG, Scandinavian and Eastern HG, Latvian HG, Latvia middle Neolithic, Lithuania early middle Neolithic and late Neolithic, Ukraine Mesolithic and Neolithic, Steppe Eneolithic, and EMBA steppe pastoralists, but not for the Lithuania Mesolithic, and European Neolithic farmer component (Figure 4). No significant allele sharing was detected between the Balts and the Anatolian Neolithic farmers.

### Ne and divergence time

**FIGURE 5:**
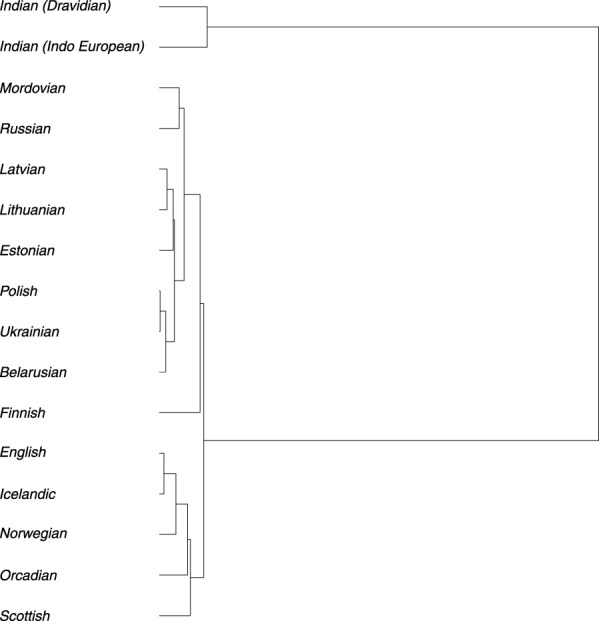
Phylogenetic tree based on the divergence time (in generations) among analyzed populations.

To reconstruct the evolutionary relationships among the Baltic, Indian (speaking Indo-European and Dravidian languages), and modern populations from Europe, effective population size (*Ne*) of each population (Supplementary Table S2, S3; Supplementary Figures S2.10, S2.11) and divergence times (Supplementary Table S2.5) among them were estimated. The estimated long-term *Ne*, calculated as the harmonic mean, was about 5,000 for Balts, with confidence intervals (CI) [4,672; 5,258]. Comparing the Ne values for populations analyzed we observed a variation from ≈10,000 in the Indian populations to 3,987 in Finnish population (Supplementary Figure S2.3). The constructed phylogenetic tree displays two main long branches which distinguish Indians from other populations in the European continent. This indicates an earlier separation that occurred many years ago. Divergence time analysis showed that Indian population separated from other European continent populations approximately 11,569 years ago on average (Supplementary Table S2.5). The branch representing populations from the European continent further divides into two smaller main branches, which can be partly explained by the language families and their branches and partly by the geographic proximity of the populations. One branch comprises Indo-European speakers of the Baltic (Lithuanian and Latvian), the West Slavic (Polish), and the East Slavic (Russian, Belarusian, Ukrainian), which belong to the Balto-Slavic branch, but it also includes speakers of Uralic languages from the Finnic (Estonian and Finnish) and the Mordvin (Mordovian) branches. The speakers of different subbranches and even families are notably intermixed here due to the areal proximity: Polish (West Slavic) shares a branch with Ukrainian (East Slavic), Lithuanian and Latvian (Indo-European) share a branch with Estonian (Uralic), and Russian (Indo-European) shares a branch with Mordovian (Uralic). The other branch forms a group of Germanic-speaking populations, where speakers of West Germanic (English, Scottish, Orcadian) and North Germanic (Icelandic, Norwegian) are intermixed. Within the first branch group, the Finnish population is initially separated from the remaining populations. Subsequently, the Mordovian and Russian populations are distinguished from the rest. Further smaller branches then separate into a group consisting of Estonian, Latvian, and Lithuanian populations and another group comprising Polish, Belarusian, and Ukrainian populations. The most recent separation occurred between Lithuanian and Latvian populations and between Polish and Ukrainian populations. The Scottish population splits from the rest in the Germanic branch first. Following that, the Orcadian and Norwegian populations are distinguished, and the most recent separation occurred between English and Icelandic populations (Figure 5).

## Discussion

The results of our present study, using increased number of the Baltic samples, confirm previous findings that the Lithuanian and Latvian populations exhibit a high level of homogeneity (Kasperavičiūtė et al., 2004; Pliss et al., 2015; Ruzgaitė et al., 2015; Urnikyte et al., 2019). The Indo-European speaking Indian population clusters closer to Lithuanian, Latvian, and other Indo-European and Uralic speaking European populations in our sample compared to the Dravidian, Austroasiatic, or Sino-Tibetan speaking Indian populations. This supports previous findings indicating that Indo-European speaking Indians exhibit a closer genetic relationship to Europeans than Dravidian, Austroasiatic, or Sino-Tibetan speakers (Bamshad et al., 2001; Thanseem et al., 2006).

Furthermore, in all PCA plots, the Indian population displays greater data dispersion compared to other populations. This observation helps explain the higher genetic diversity observed within the Indian population (Majumder, 1998; Pugach and Stoneking, 2015). When considering other world populations, the Indian population appears to be genetically closer to European populations of our samples, including Lithuania and Latvia, than to African or East Asian populations. This finding is supported by shared genetic drift with Europeans (Bose et al., 2021). However, when conducting PCA analysis on the Baltic, Indians, and samples from ancient and recent times across the Eurasian continent, Lithuanians and Latvians did not exhibit a higher genetic relationship with Indo-European speaking Indians compared to other populations. This demonstrates that the Baltic speaking populations (within the Balto-Slavic branch) and the Indo-European-speaking Indian populations (of the Indo-Iranian branch) did not share an exclusive ancestor, and this is in line with the linguistic interpretation that does not support the tentative Indo-Slavic node in the evolution of the Indo-European language family, as discussed in the Introduction. We may also add that the results of PCA analysis demonstrate that the Indo-European speaking populations from the European continent in our (limited) sample remain equally genetically similar to the Indo-European speaking Indians.

PCA and f3-statistics analysis on ancient populations revealed that the Balts and other neighboring modern populations from the European continent display a closer genetic relationship to populations from the ancient Steppe region (from the Early-Middle and Middle-Late Bronze Age) compared to the populations from the ancient Anatolian region. These findings align with the steppe hypothesis, which suggests that the major spread of Indo-Europeans to Europe was from the Pontic Steppe (Gimbutas, 1970; Mallory, 1989; Anthony, 2007) or the hybrid origin model which presupposes a secondary Indo-European homeland in the steppe region (Heggarty et al., 2023), rather than the Anatolian hypothesis alone (Renfrew, 1987; Bellwood, 2005).

Furthermore, an analysis of the population’s genetic structure using the admixture method revealed that the genetic composition of the Indian population differs from that of other populations across the Eurasian continent, both ancient and modern. However, once again, the Indo-European speaking part of the Indian population showed more similar genetic composition to other European populations compared to Dravidians.

The divergence time analysis indicates that Indian population separated from other European continent populations approximately 11,569 years ago on average. They separated from Lithuanians 12,673 years ago and from Latvians 12,969 years ago. The most recent divergence for the Indian population, in the context of European populations, occurred with the Mordovian population approximately 11,195 years ago. This period may be associated with the retreat of the last glacier, as well as the separation and dispersal of Indo-European languages across the Eurasian continent.

In conclusion, these results largely confirm previous findings reported by other authors in the field of population genetic studies or, at the very least, do not conflict with them. The Indo-European speaking Indian population exhibits a higher similarity to Europeans in both genetic structure analysis methods (PCA and admixture), as previously discussed in literature (Bamshad et al., 2001; Thanseem et al., 2006; Bose et al., 2021). However, as already mentioned above, the applied methods did not reveal that the Balts population is more genetically similar to Indians than other Indo-European speaking European populations. This work represents the first attempt in the field of population genetic studies to investigate the genetic relationship between the Baltic and the Indian populations using autosomal markers. Additionally, the study included a large number of samples from the investigated Baltic and Indian populations, ensuring diversity within the datasets.

The study does have several limitations that should be considered. First, while the sample size was large, it may not fully capture the entire genetic diversity of the Baltic and Indian populations, potentially limiting the generalizability of the findings. Second, the study focused on autosomal markers, which, while informative, may not provide a complete picture of genetic relationships. Other genetic markers, such as mitochondrial DNA or Y-chromosome data, could offer additional insights.

## Data Availability

The datasets presented in this study can be found in online repositories. The names of the repository/repositories and accession number(s) can be found below: https://www.ebi.ac.uk/ena/browser/view/PRJEB25864; PRJEB25864. Additional data information is available in Supplementary Table S1.
