# Supplementary figures and images for: Populations of Latvia and Lithuania in the context of some Indo-European and non-Indo-European speaking populations of Europe and India: insights from genetic structure analysis
Source: Front Genet. 2024 Nov 20;15:1493270. doi: 10.3389/fgene.2024.1493270 (PMC11614816; doi:10.3389/fgene.2024.1493270)

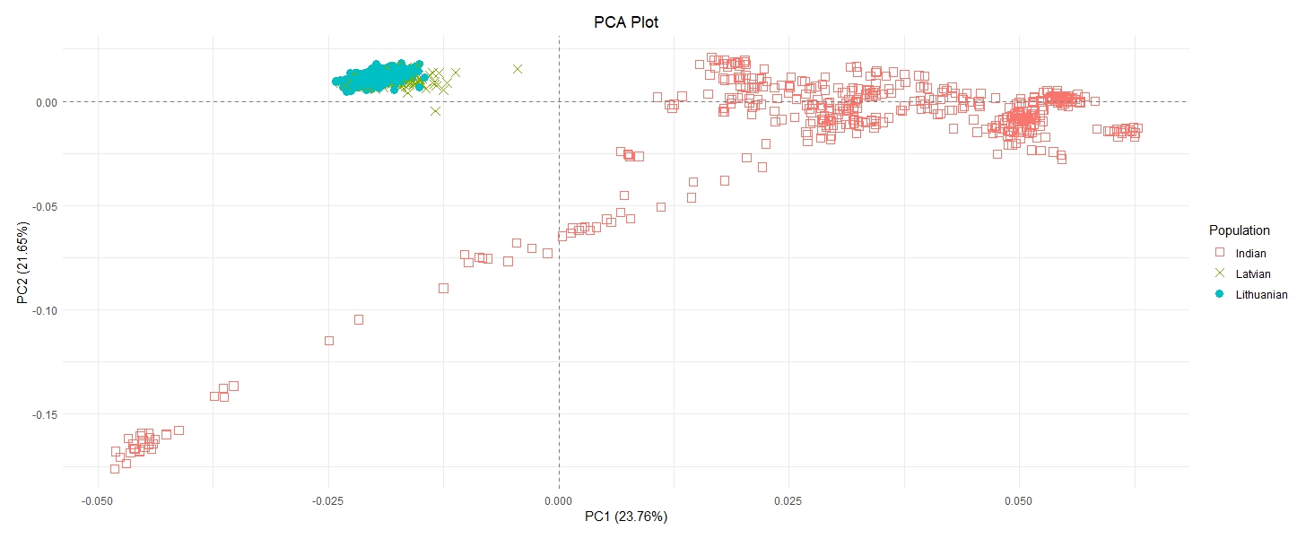

Supplement: Supplementary file 1 [file DataSheet1.ZIP › Supplementary figure 2.1.png]

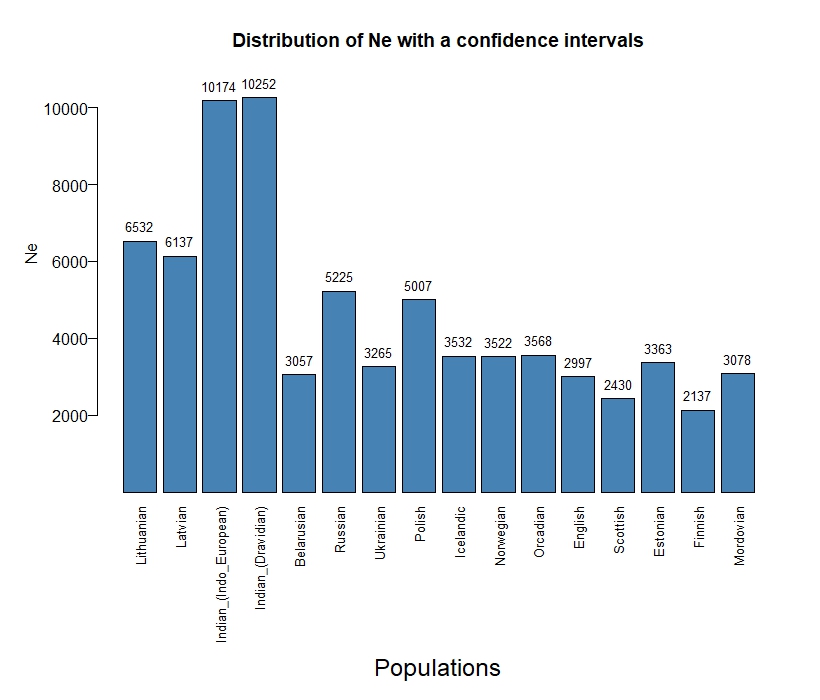

Supplement: Supplementary file 1 [file DataSheet1.ZIP › Supplementary figure 2.10.jpeg]

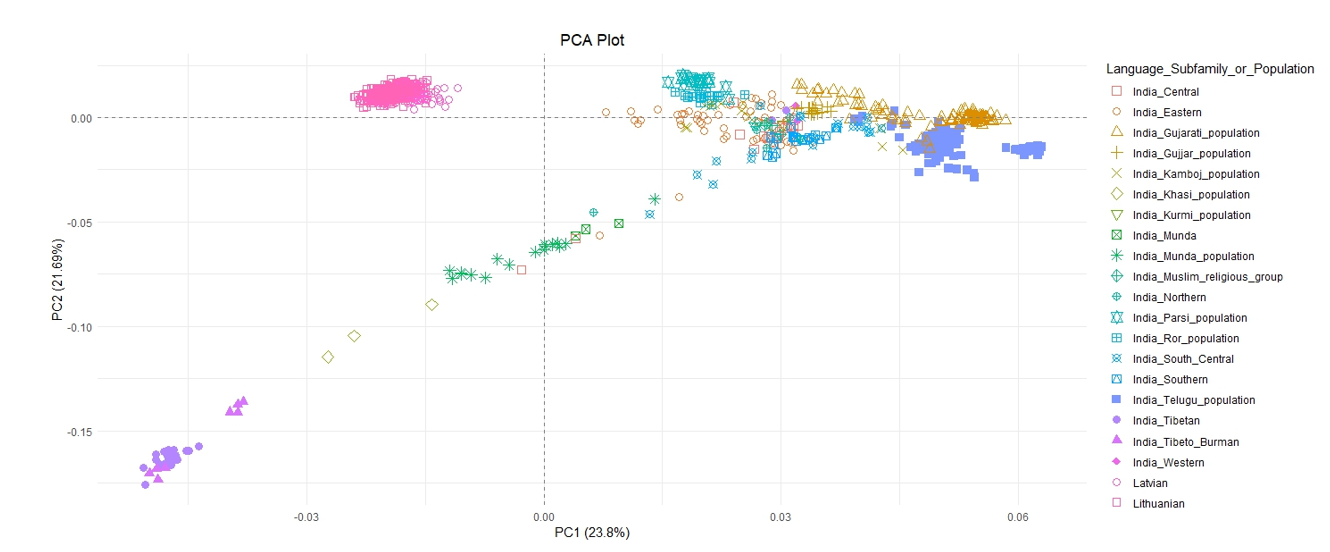

Supplement: Supplementary file 1 [file DataSheet1.ZIP › Supplementary figure 2.2.png]

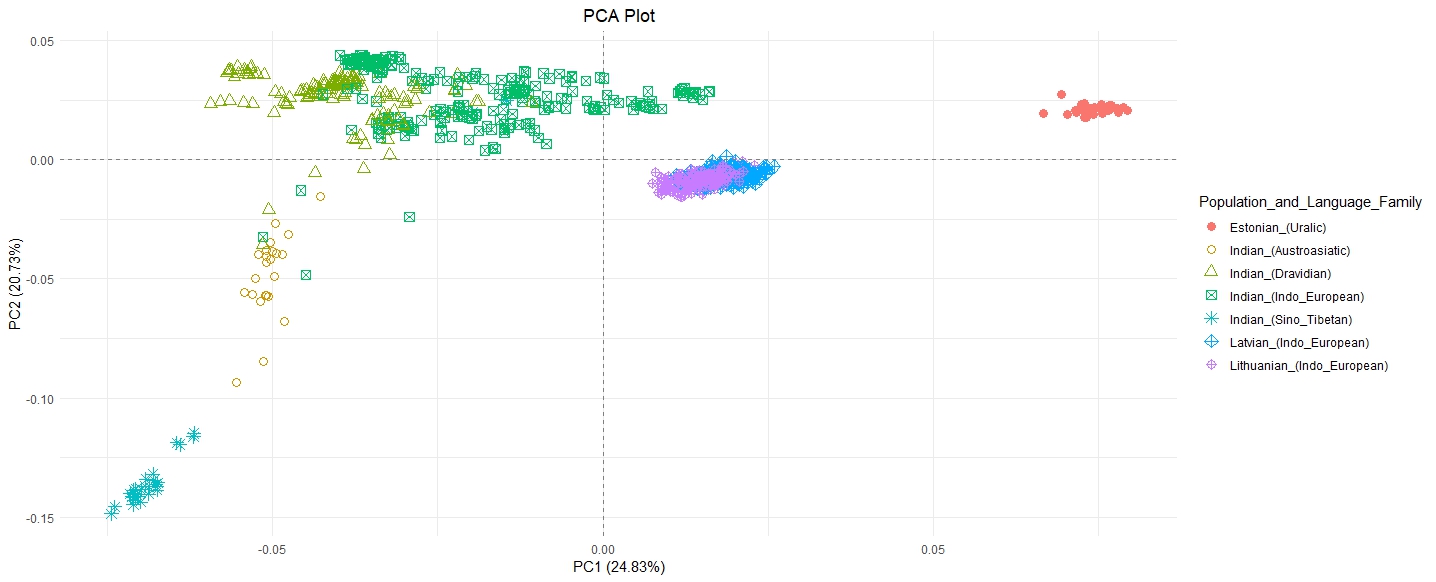

Supplement: Supplementary file 1 [file DataSheet1.ZIP › Supplementary figure 2.3.jpeg]

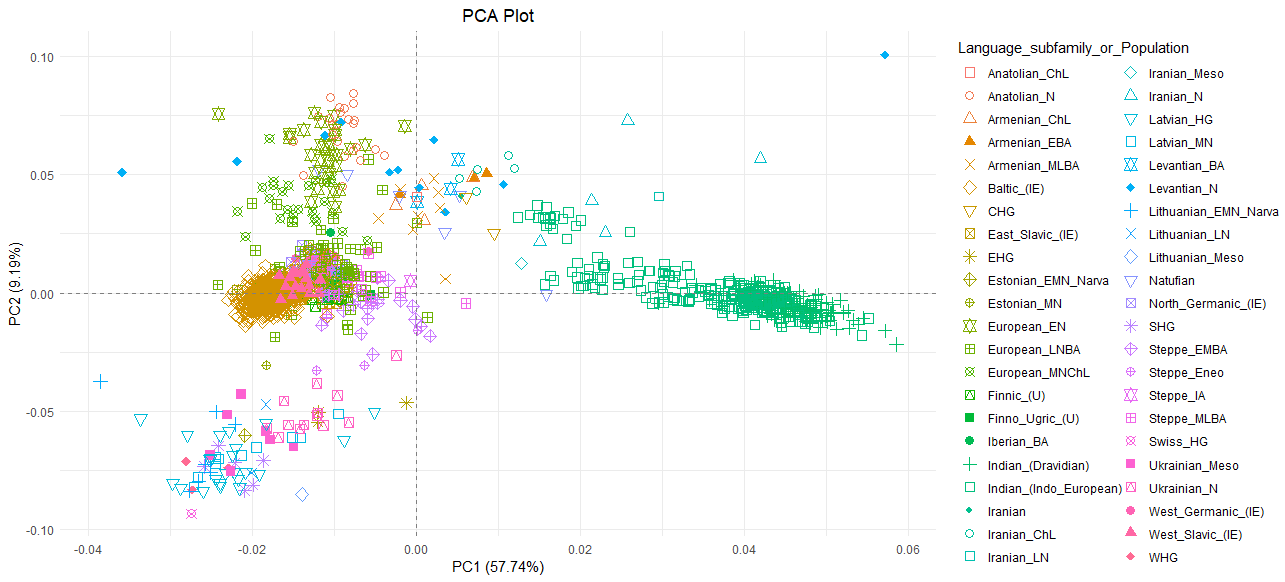

Supplement: Supplementary file 1 [file DataSheet1.ZIP › Supplementary figure 2.4.png]

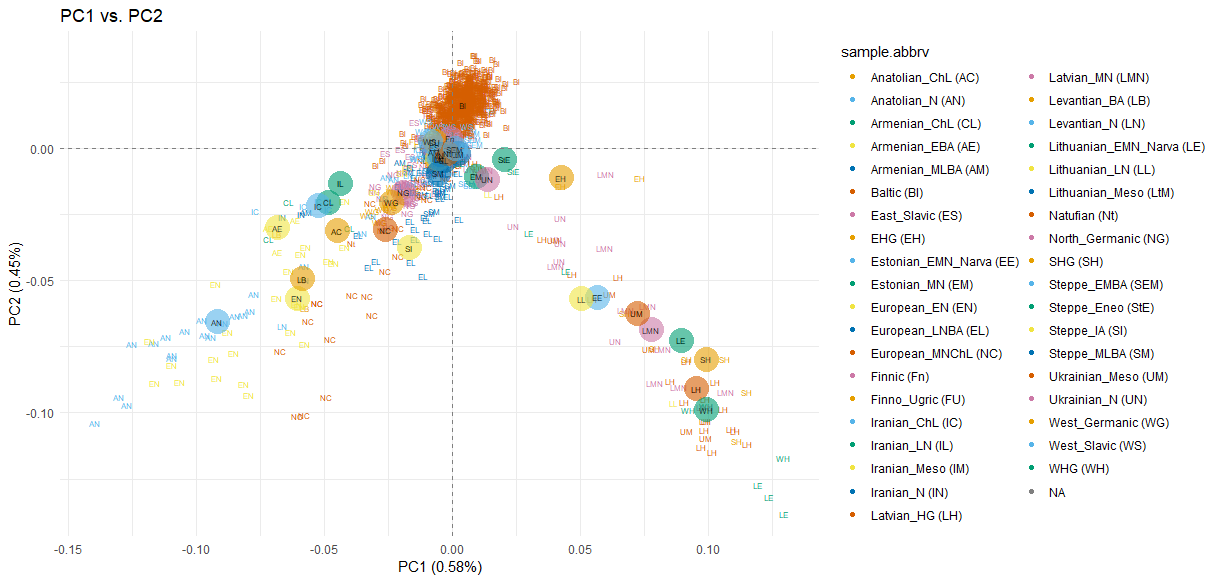

Supplement: Supplementary file 1 [file DataSheet1.ZIP › Supplementary figure 2.5.png]

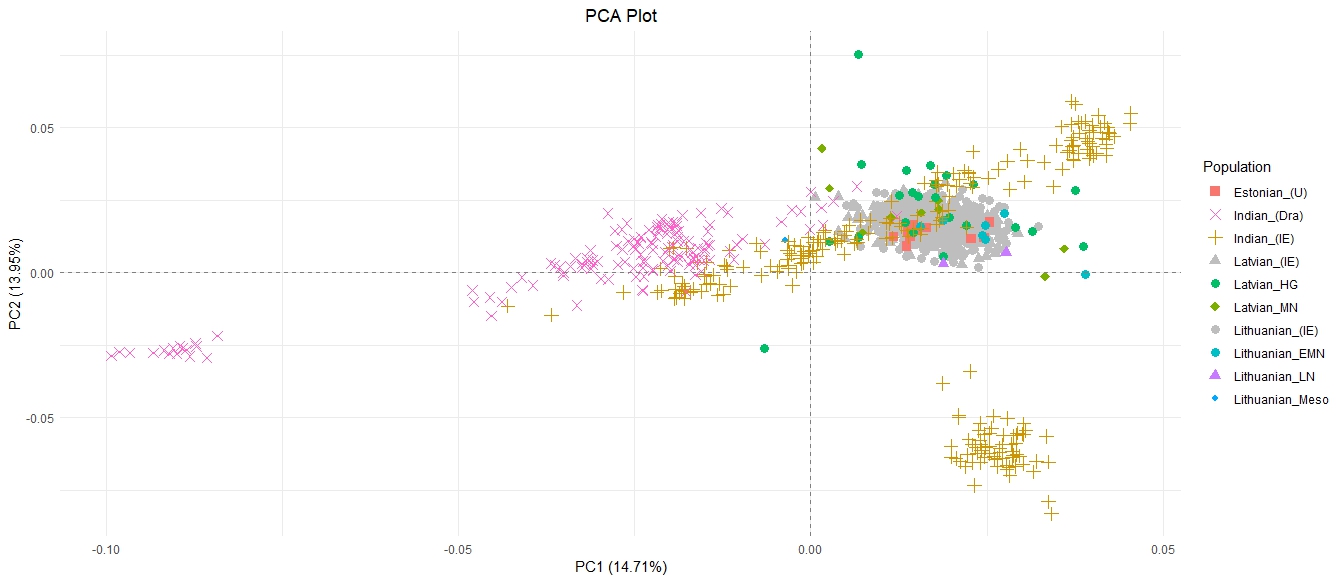

Supplement: Supplementary file 1 [file DataSheet1.ZIP › Supplementary figure 2.6.jpeg]

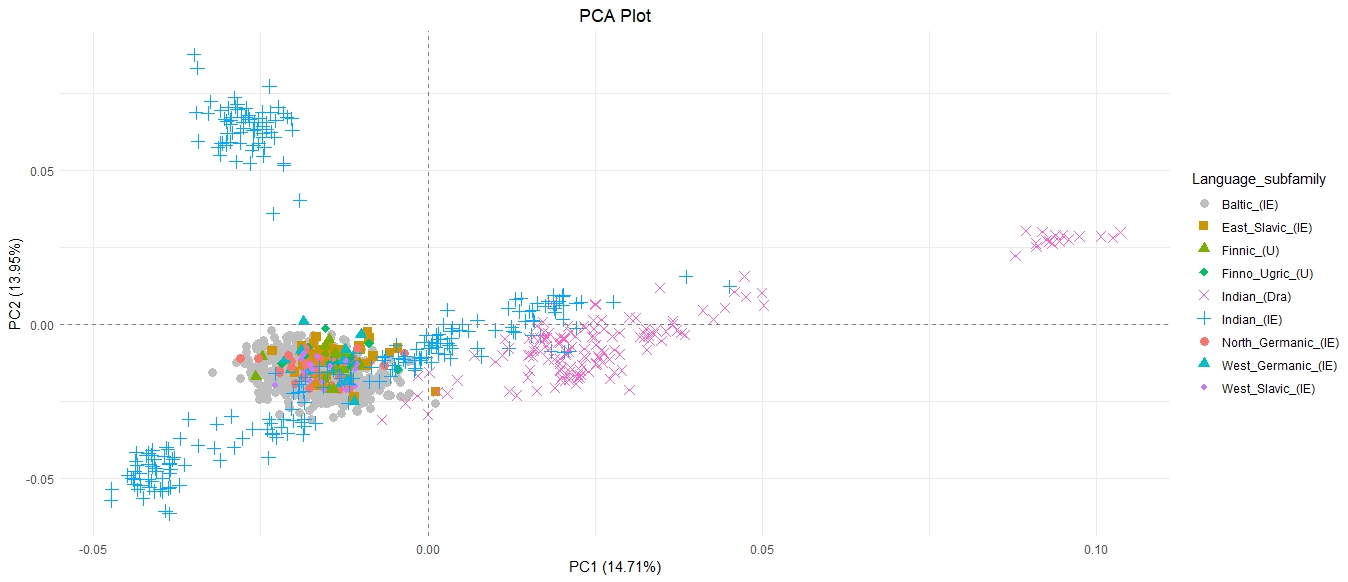

Supplement: Supplementary file 1 [file DataSheet1.ZIP › Supplementary figure 2.7.jpeg]

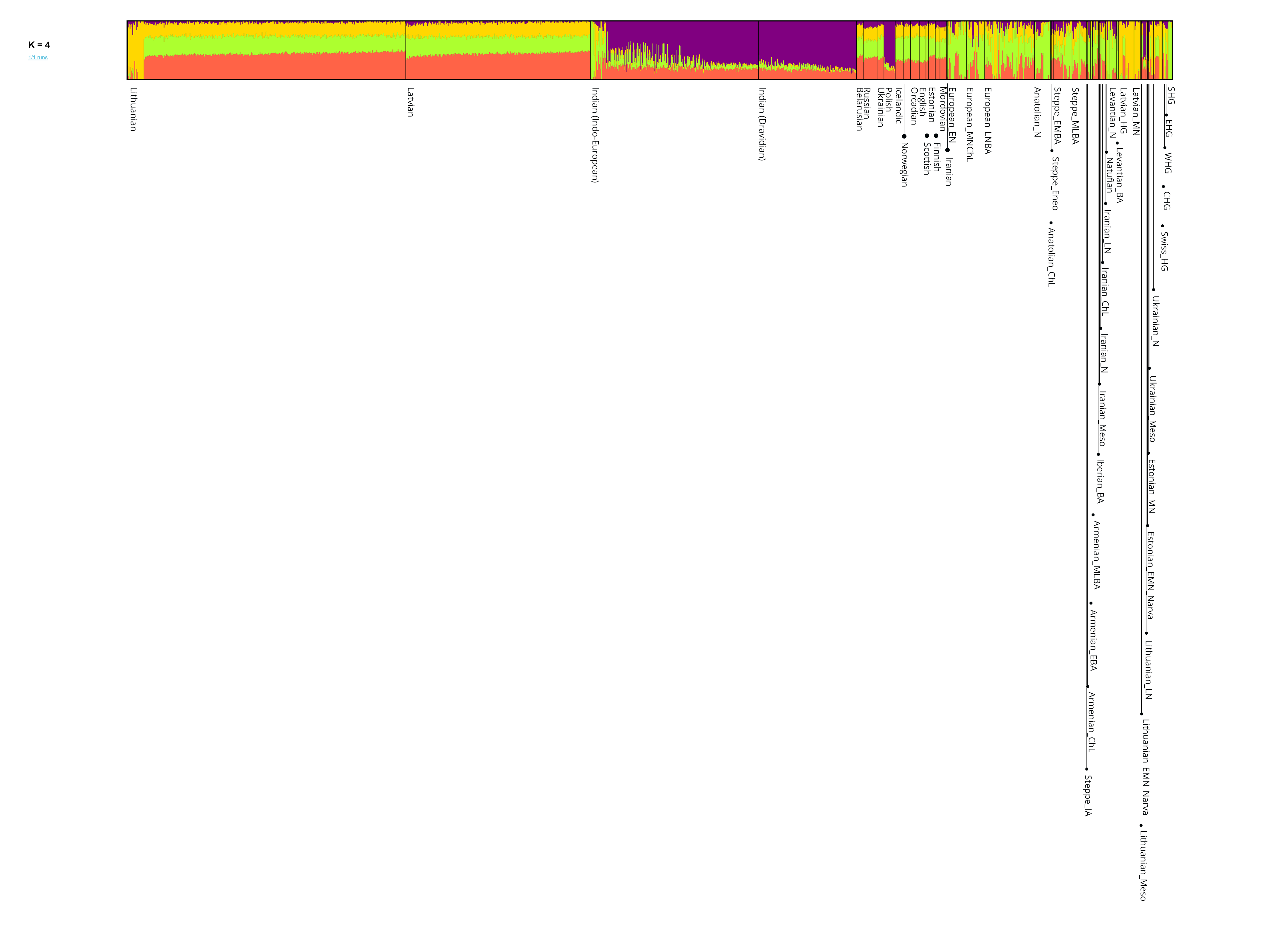

Supplement: Supplementary file 1 [file DataSheet1.ZIP › Supplementary figure 2.8.png]

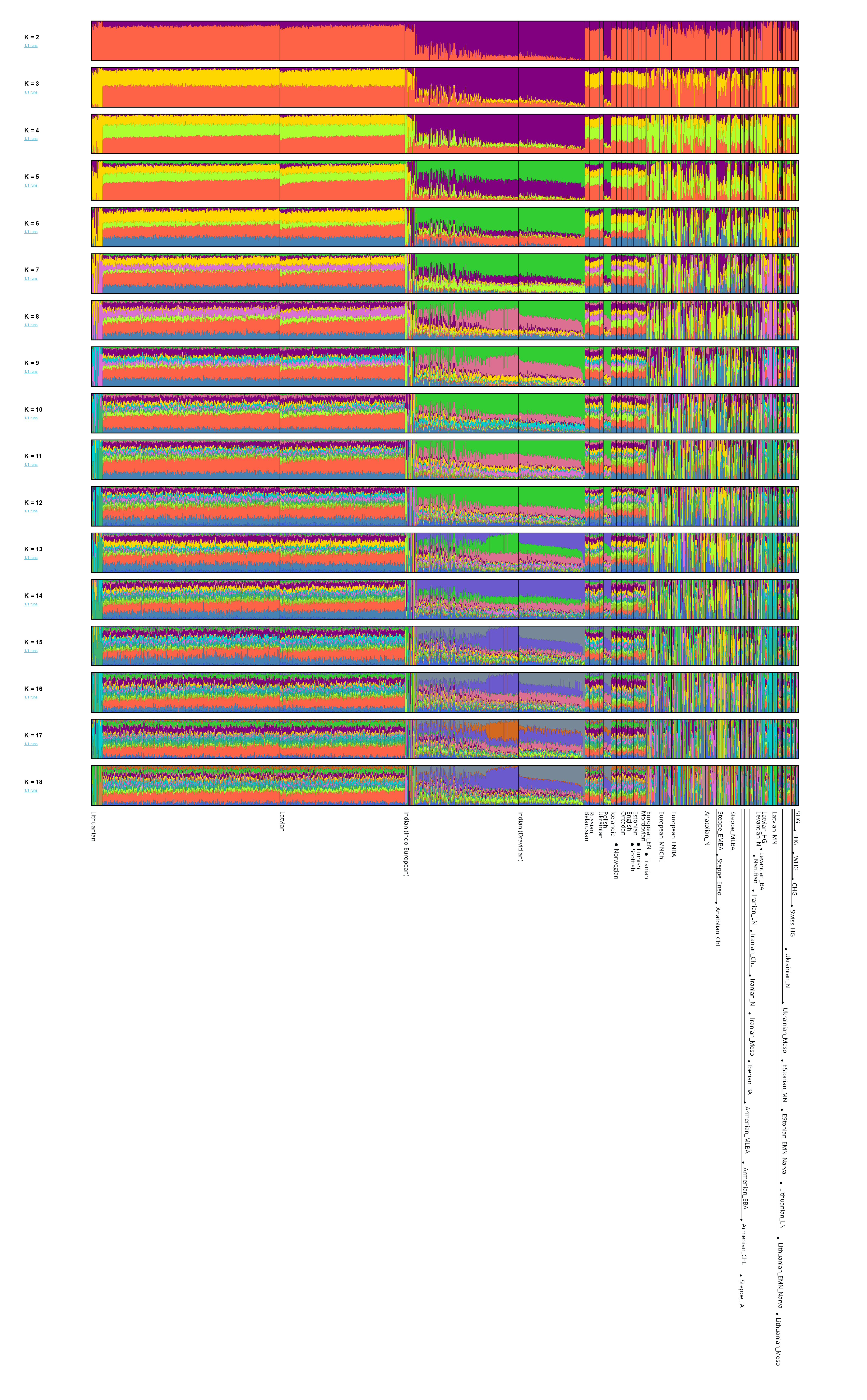

Supplement: Supplementary file 1 [file DataSheet1.ZIP › Supplementary figure 2.9.png]

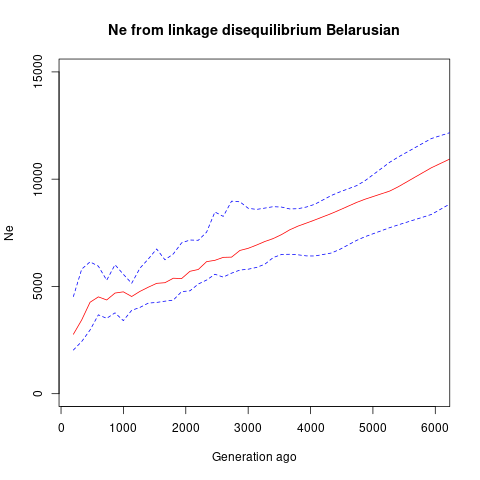

Supplement: Supplementary file 1 [file DataSheet1.ZIP › Supplementary figures 2.11/Belarusian_Med_plot.png]

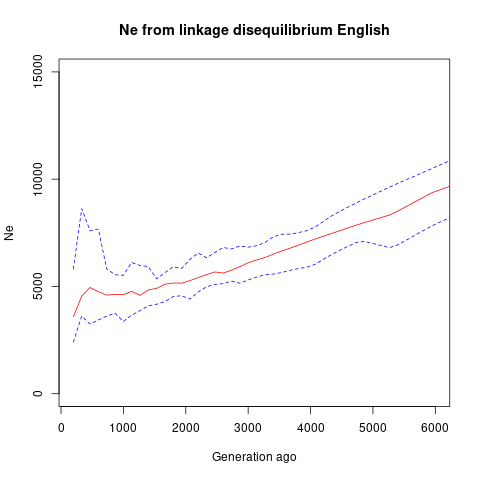

Supplement: Supplementary file 1 [file DataSheet1.ZIP › Supplementary figures 2.11/English_Med_plot.png]

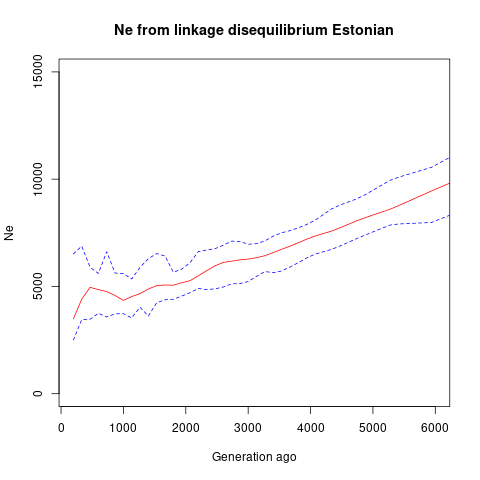

Supplement: Supplementary file 1 [file DataSheet1.ZIP › Supplementary figures 2.11/Estonian_Med_plot.png]

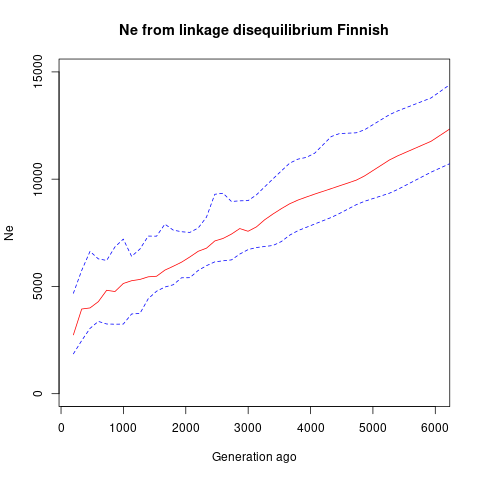

Supplement: Supplementary file 1 [file DataSheet1.ZIP › Supplementary figures 2.11/Finnish_Med_plot.png]

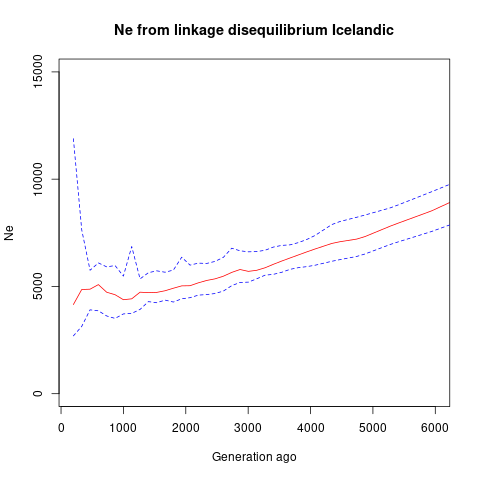

Supplement: Supplementary file 1 [file DataSheet1.ZIP › Supplementary figures 2.11/Icelandic_Med_plot.png]

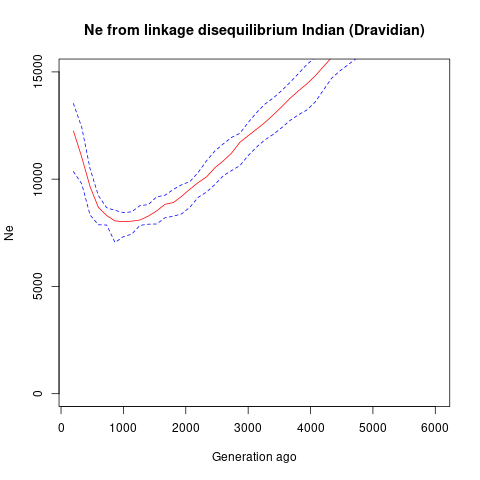

Supplement: Supplementary file 1 [file DataSheet1.ZIP › Supplementary figures 2.11/Ind_Dra_Med_plot.png]

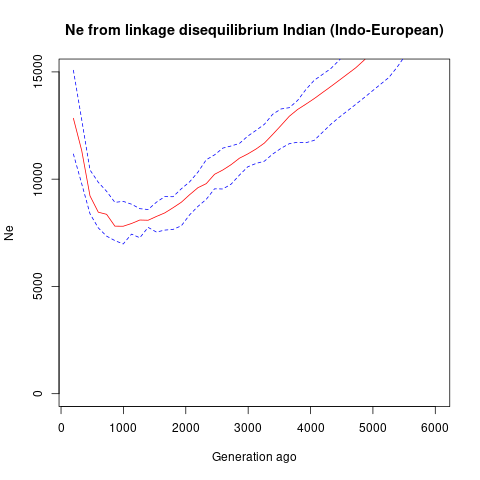

Supplement: Supplementary file 1 [file DataSheet1.ZIP › Supplementary figures 2.11/Ind_IE_Med_plot.png]

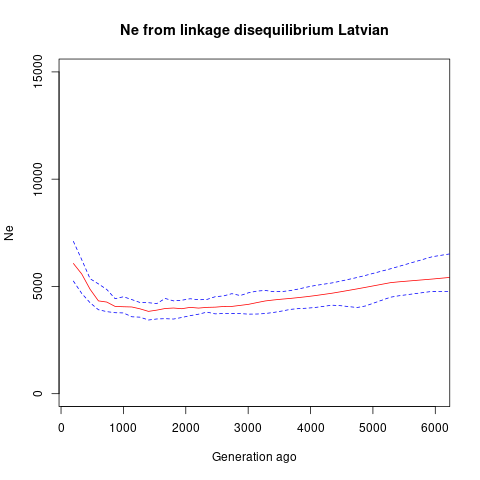

Supplement: Supplementary file 1 [file DataSheet1.ZIP › Supplementary figures 2.11/Latvian_Med_plot.png]

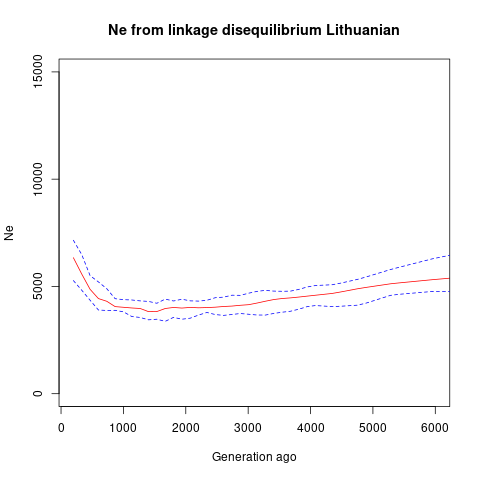

Supplement: Supplementary file 1 [file DataSheet1.ZIP › Supplementary figures 2.11/Lithuanian_Med_plot.png]

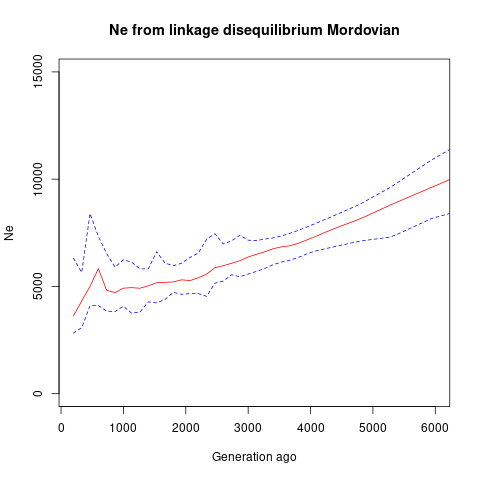

Supplement: Supplementary file 1 [file DataSheet1.ZIP › Supplementary figures 2.11/Mordovian_Med_plot.png]

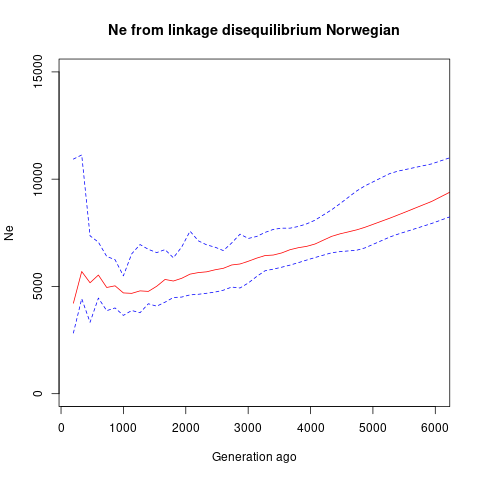

Supplement: Supplementary file 1 [file DataSheet1.ZIP › Supplementary figures 2.11/Norwegian_Med_plot.png]

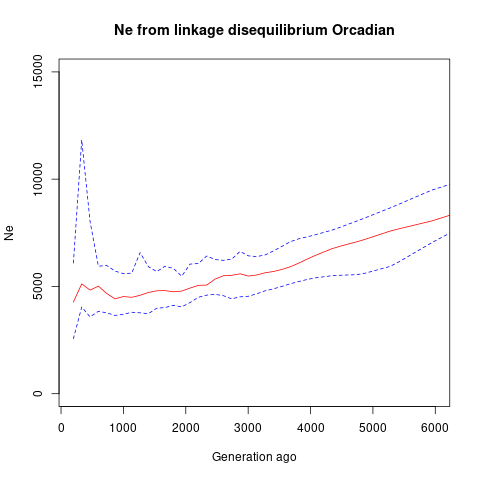

Supplement: Supplementary file 1 [file DataSheet1.ZIP › Supplementary figures 2.11/Orcadian_Med_plot.png]

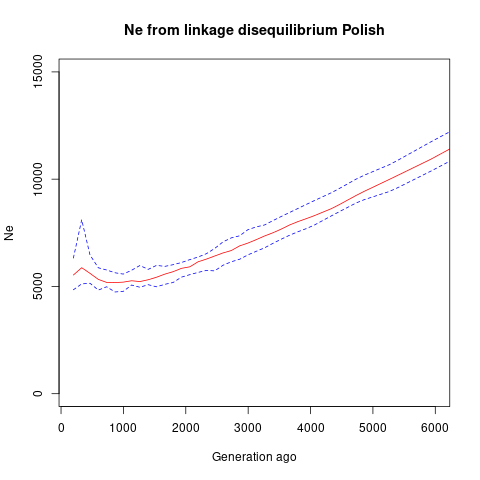

Supplement: Supplementary file 1 [file DataSheet1.ZIP › Supplementary figures 2.11/Polish_Med_plot.png]

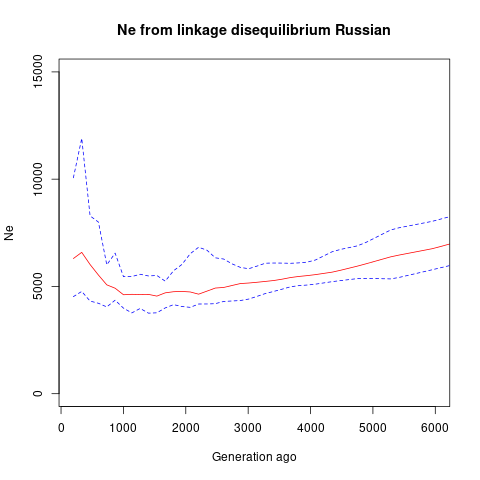

Supplement: Supplementary file 1 [file DataSheet1.ZIP › Supplementary figures 2.11/Russian_Med_plot.png]

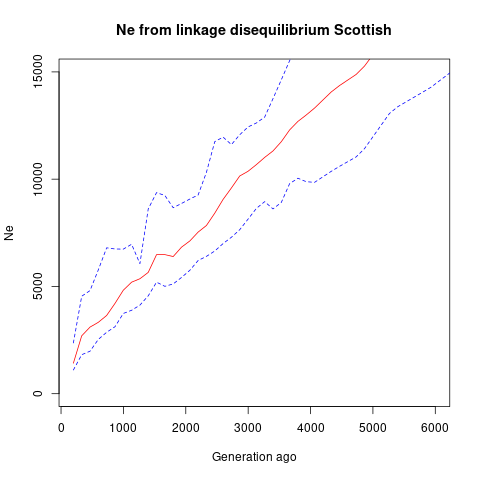

Supplement: Supplementary file 1 [file DataSheet1.ZIP › Supplementary figures 2.11/Scottish_Med_plot.png]

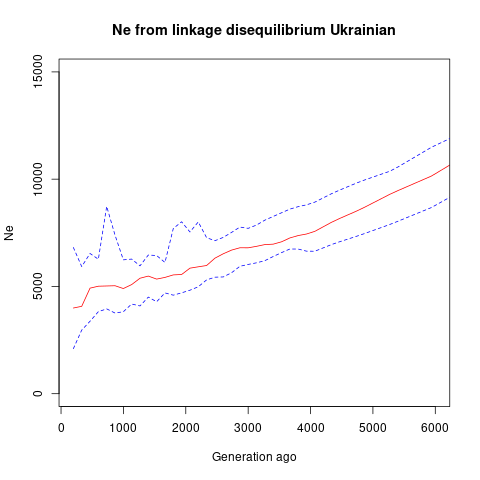

Supplement: Supplementary file 1 [file DataSheet1.ZIP › Supplementary figures 2.11/Ukrainian_Med_plot.png]
