# Supplementary material for: Populations of Latvia and Lithuania in the context of some Indo-European and non-Indo-European speaking populations of Europe and India: insights from genetic structure analysis
Source: Front Genet. 2024 Nov 20;15:1493270. doi: 10.3389/fgene.2024.1493270 (PMC11614816; doi:10.3389/fgene.2024.1493270)
Supplement: Supplementary file 2 [file DataSheet2.ZIP › Supplementary table 1.1.pdf]

Country	Dialect region	Number of samples
Lithuania	Žemaitija	194
	Aukštaitija	213
Latvia	Courland	60
	Latgale	51
	Semigallia	23
	Vidzeme	143
