# Supplementary material for: Populations of Latvia and Lithuania in the context of some Indo-European and non-Indo-European speaking populations of Europe and India: insights from genetic structure analysis
Source: Front Genet. 2024 Nov 20;15:1493270. doi: 10.3389/fgene.2024.1493270 (PMC11614816; doi:10.3389/fgene.2024.1493270)
Supplement: Supplementary file 2 [file DataSheet2.ZIP › Supplementary table 1.2.pdf]

Ethnic group	Number of samples in ethnic group	Language family	Language subfamily or population	Number of samples in language subfamily/population
Brahmins from Tamil Nadu	2	Dravidian	South-Central	20
Chenchus	4			
Gond	4			
Velamas	10			
Lambadi	1	Dravidian	Central	21
Dhurwa	1			
Kol	16	Indo-European		
Tharus	3			
Hakkikipikki	4	Dravidian	Southern	23
Kurumba	4			
Piramalai Kallars	8			
Pulliyar	5			
Tamil Nadu Scheduled Caste	2	Indo-European	Southern	
Kshatriya	7	Indo-European	Eastern	57
Bengali	1			
Bhunjia	1			
Brahmins from Uttar Pradesh	7			
Chamar	10			
Dharkars	12			
Dusadh	10			
Kanjars	9			
Brahmins from Uttaranchal	1	Indo-European	Northern	6
Uttar Pradesh Scheduled Caste	5			
Meena	1	Indo-European	Western	2
Meghawal	1			

Garo	4	Sino-Tibetan	Tibeto-Burman	8
Naga	4			
Hmar	4	Sino-Tibetan	Tibetan	22
Kom	2			
Kuki	6			
Mizo	5			
Naga	1			
Nyishi	4			
Muslim	5	Religious group (Indo-European)	Muslim religious group	5
Kurmi	1	Indo-European	Kurmi population	1
Gujjar	15	Indo-European	Gujjar population	15
Ror	15	Indo-European	Ror population	15
Gujarati	103	Indo-European	Gujarati population	103
Parsi	19	Indo-European	Parsi population	19
Kamboj	14	Indo-European	Kamboj population	14
Khasi	3	Austroasiatic	Khasi population	3
Telugu	102	Dravidian	Telugu population	102
Mawasi	1	Austroasiatic	Munda population	20
Asur	2	Austroasiatic		
Ho	5	Austroasiatic		
Santhal	1	Austroasiatic		
Bonda	4	Austroasiatic		
Gadaba	1	Austroasiatic		
Juang	2	Austroasiatic		
Kharia	2	Austroasiatic		
Savara	2	Austroasiatic		
