# Supplementary material for: Populations of Latvia and Lithuania in the context of some Indo-European and non-Indo-European speaking populations of Europe and India: insights from genetic structure analysis
Source: Front Genet. 2024 Nov 20;15:1493270. doi: 10.3389/fgene.2024.1493270 (PMC11614816; doi:10.3389/fgene.2024.1493270)
Supplement: Supplementary file 2 [file DataSheet2.ZIP › Supplementary table 1.3.pdf]

Geographic region	Population	Abbreviation	Number of samples
Africa	Yoruba in Ibadan, Nigeria	YRI	108
	Luhya in Webuye, Kenya	LWK	99
	Gambian in Western Divisions in the Gambia	GWD	113
	Mende in Sierra Leone	MSL	85
	Esan in Nigeria	ESN	99
Europe	Utah Residents with Northern and Western European Ancestry	CEU	99
	Toscani in Italia	TSI	107
	Finnish in Finland	FIN	99
	British in England and Scotland	GBR	91
	Estonians from Estonia	Estonians	36
East Asia	Han Chinese in Bejing, China	CHB	103
	Japanese in Tokyo, Japan	JPT	104
	Southern Han Chinese, China	CHS	105
	Chinese Dai in Xishuangbanna, China	CDX	93
	Kinh in Ho Chi Minh City, Vietnam	KHV	99
South Asia	Punjabi from Lahore, Pakistan	PJL	96
	Bengali from Bangladesh	BEB	86
	Sri Lankan Tamil from the UK	STU	102
