# Supplementary material for: Populations of Latvia and Lithuania in the context of some Indo-European and non-Indo-European speaking populations of Europe and India: insights from genetic structure analysis
Source: Front Genet. 2024 Nov 20;15:1493270. doi: 10.3389/fgene.2024.1493270 (PMC11614816; doi:10.3389/fgene.2024.1493270)
Supplement: Supplementary file 2 [file DataSheet2.ZIP › Supplementary table 1.4.pdf]

Period	Age	Population	Abbreviation	Number of Samples
Late Paleolithic (Old Stone Age)	~ 30,000 to 10,000 BCE	Natufian	-	6
		Swiss Hunter Gatherers	Switzerland HG	1
Mesolithic (Middle Stone Age)	~ 10,000 to 7000 BCE	Iranian Mesolithic	Iranian Meso	1
		Ukrainian Mesolithic	Ukrainian Meso	7
		Lithuanian Mesolithic	Lithuanian Meso	1
		Caucasian Hunter Gatherers	CHG	2
Neolithic (New Stone Age)	~ 7000 to 3000 BCE, varying by region	European Early Neolithic	European EN	29
		Latvian Hunter Gatherers	Latvian HG	23
		Scandinavian Hunter Gatherers	SHG	6
		Eastern Hunter Gatherers	EHG	3
		Western Hunter Gatherers	WHG	3
		Levantian Neolithic	Levantian N	13
		Iranian Neolithic	Iranian N	5
		Anatolian Neolithic	Anatolian N	24
		Ukrainian Neolithic	Ukrainian N	13
		Lithuanian Early – Middle Neolithic (Narva culture)	Lithuanian EMN Narva	7

		Estonian Early – Middle Neolithic (Narva culture)	Estonian EMN Narva	1
		Latvian Middle Neolithic	Latvian MN	10
		Estonian Middle Neolithic	Estonian MN	1
		European Middle Neolithic - Chalcolithic	European MNChL	27
		Iran Chalcolithic	Iranian ChL	5
		Anatolian Chalcolithic	Anatolian ChL	1
		Armenian Chalcolithic	Armenian ChL	5
		Steppe Eneolithic	Steppe Eneo	3
		Lithuanian Late Neolithic	Lithuanian LN	2
		Iranian Late Neolithic	Iranian LN	1
Bronze age	~ 3300 to 1200 BCE	European Late Neolithic – Bronze Age	European LNBA	75
		Armenian Early Bronze Age	Armenian EBA	3
		Iberian Bronze Age	Iberian BA	1
		Levantian Bronze Age	Levantian BA	3
		Armenian Middle – Late Bronze Age	Armenian MLBA	9
		Steppe Early – Middle Bronze Age	Steppe EMBA	28

		Steppe Middle – Late Bronze Age	Steppe MLBA	22
Iron Age	~ 1200 BCE	Steppe Iron Age	Steppe IA	1
Recent times		Finnish	-	7
		Icelandic	-	12
		Iranian	-	1
		Lithuanian (Žemaitija region)	-	10
		Mordovian	-	10
		Norwegian	-	11
		Orcadian	-	13
		Polish	-	17
		Russian	-	22
		Scottish	-	4
		Ukrainian	-	9
		Belarusian	-	10
		English	-	10
		Estonian	-	10
