# Supplementary material for: Populations of Latvia and Lithuania in the context of some Indo-European and non-Indo-European speaking populations of Europe and India: insights from genetic structure analysis
Source: Front Genet. 2024 Nov 20;15:1493270. doi: 10.3389/fgene.2024.1493270 (PMC11614816; doi:10.3389/fgene.2024.1493270)
Supplement: Supplementary file 2 [file DataSheet2.ZIP › Supplementary table 2.1.pdf]

Populations	Number of individuals	Average kinship coefficient	Individuals with kinship >0.0884	Average inbreeding coefficient (F)	Individuals with F >0,0156
Lithuania	416	0,0008	2 (1 removed)	0,0034	8 (2,16%)
Latvia	287	0,0006	0	0,0034	8 (2,78%)
India	456	0,0005	368	0,0239	193 (42,32%)
