# Supplementary material for: Populations of Latvia and Lithuania in the context of some Indo-European and non-Indo-European speaking populations of Europe and India: insights from genetic structure analysis
Source: Front Genet. 2024 Nov 20;15:1493270. doi: 10.3389/fgene.2024.1493270 (PMC11614816; doi:10.3389/fgene.2024.1493270)
Supplement: Supplementary file 2 [file DataSheet2.ZIP › Supplementary table 2.2.pdf]

**K number**	**CV value**
2	0.63113
3	0.63028
4	0.63000
5	0.63018
6	0.63176
7	0.63578
8	0.63527
9	0.63910
10	0.64510
11	0.64417
12	0.65156
13	0.64701
14	0.65455
15	0.65218
16	0.66087
17	0.65882
18	0.67488
