# Supplementary material for: Populations of Latvia and Lithuania in the context of some Indo-European and non-Indo-European speaking populations of Europe and India: insights from genetic structure analysis
Source: Front Genet. 2024 Nov 20;15:1493270. doi: 10.3389/fgene.2024.1493270 (PMC11614816; doi:10.3389/fgene.2024.1493270)
Supplement: Supplementary file 2 [file DataSheet2.ZIP › Supplementary table 2.3.pdf]

| Population | Number of samples | Ne | 95 % CI |
|---|---|---|---|
| Lithuanian | 417 | 5,100 | [4,672; 5,258] |
| Latvian | 277 | 5,014 | [4,678; 5,168] |
| Indian (Indo-European) | 252 | 10,174 | [9,363; 10,712] |
| Indian (Dravidian) | 147 | 10,252 | [9,702; 10,846] |
| Belarusian | 10 | 4,015 | [3,561; 4,403] |
| Russian | 22 | 5,872 | [5,138; 6,436] |
| Ukrainian | 9 | 4,602 | [4,035; 5,320] |
| Polish | 17 | 5,726 | [5,390; 5,960] |
| Icelandic | 12 | 4,742 | [4,168; 5,363] |
| Norwegian | 11 | 5,141 | [4,248; 5,859] |
| Orcadian | 13 | 4,866 | [4,315; 5,385] |
| English | 10 | 4,719 | [4,038; 5,723] |
| Scottish | 4 | 2,478 | [2,166; 2,942] |
| Estonian | 10 | 4,421 | [4,021; 4,863] |
| Finnish | 7 | 3,987 | [3,135; 4,638] |
| Mordovian | 10 | 4,697 | [4,367; 5,302] |
