# Supplementary material for: Populations of Latvia and Lithuania in the context of some Indo-European and non-Indo-European speaking populations of Europe and India: insights from genetic structure analysis
Source: Front Genet. 2024 Nov 20;15:1493270. doi: 10.3389/fgene.2024.1493270 (PMC11614816; doi:10.3389/fgene.2024.1493270)
Supplement: Supplementary file 2 [file DataSheet2.ZIP › Supplementary table 2.4.pdf]

Polish	Ukrainian	Russian	Belarusian	Indian (Dravidian)	Indian (Indo-European)	Latvian	Lithuanian	POPULATION
							0.0000	**Lithuanian**
						0.0000	0.0010	**Latvian**
					0.0000	0.0336	0.0326	**Indian (Indo-European)**
				0.0000	0.0031	0.0432	0.0423	**Indian (Dravidian)**
			0.0000	0.0443	0.0330	0.0019	0.0012	**Belarusian**
		0.0000	0.0027	0.0378	0.0275	0.0037	0.0037	**Russian**
	0.0000	0.0021	0.0015	0.0440	0.0326	0.0032	0.0030	**Ukrainian**
0.0000	0.0001	0.0023	0.0003	0.0422	0.0310	0.0014	0.0010	**Polish**
0.0035	0.0050	0.0049	0.0056	0.0450	0.0335	0.0065	0.0062	**Icelandic**
0.0045	0.0056	0.0060	0.0064	0.0459	0.0340	0.0073	0.0065	**Norwegian**
0.0055	0.0058	0.0070	0.0067	0.0460	0.0345	0.0089	0.0084	**Orcadian**
0.0032	0.0039	0.0046	0.0043	0.0436	0.0321	0.0069	0.0062	**English**
0.0063	0.0066	0.0085	0.0060	0.0499	0.0379	0.0086	0.0083	**Scottish**
0.0018	0.0032	0.0034	0.0021	0.0451	0.0341	0.0018	0.0021	**Estonian**
0.0061	0.0061	0.0037	0.0059	0.0459	0.0351	0.0071	0.0073	**Finnish**
0.0029	0.0037	0.0025	0.0029	0.0406	0.0297	0.0043	0.0041	**Mordovian**

Mordovian	Finnish	Estonian	Scottish	English	Orcadian	Norwegian	Icelandic
							0.0000
						0.0000	0.0024
					0.0000	0.0044	0.0039
				0.0000	0.0031	0.0021	0.0006
			0.0000	0.0038	0.0071	0.0068	0.0046
		0.0000	0.0064	0.0042	0.0078	0.0049	0.0054
	0.0000	0.0043	0.0104	0.0072	0.0100	0.0077	0.0074
0.0000	0.0077	0.0031	0.0085	0.0057	0.0079	0.0065	0.0061
