# Supplementary material for: Populations of Latvia and Lithuania in the context of some Indo-European and non-Indo-European speaking populations of Europe and India: insights from genetic structure analysis
Source: Front Genet. 2024 Nov 20;15:1493270. doi: 10.3389/fgene.2024.1493270 (PMC11614816; doi:10.3389/fgene.2024.1493270)
Supplement: Supplementary file 2 [file DataSheet2.ZIP › Supplementary table 2.5.pdf]

Polish	Ukrainian	Russian	Belarusian	Indian (Dravidian)	Indian (Indo-European)	Latvian	Lithuanian	POPULATION
							0	**Lithuanian**
						0	253	**Latvian**
					0	12,969	12,673	**Indian (Indo-European)**
				0	1,583	16,858	16,580	**Indian (Dravidian)**
			0	16,171	11,919	426	280	**Belarusian**
		0	669	15,548	11,181	1,011	1,021	**Russian**
	0	554	333	16,726	12,248	766	719	**Ukrainian**
0	18	656	84	17,226	12,531	384	265	**Polish**
912	1,180	1,306	1,224	17,270	12,709	1,585	1,525	**Icelandic**
1,226	1,369	1,663	1,475	18,066	13,226	1,858	1,676	**Norwegian**
1,455	1,387	1,877	1,483	17,799	13,213	2,201	2,092	**Orcadian**
826	902	1,234	945	16,673	12,158	1,676	1,529	**English**
1,306	1,178	1,775	970	16,294	12,210	1,623	1,588	**Scottish**
447	731	883	438	16,927	12,667	416	502	**Estonian**
1,493	1,304	922	1,176	16,724	12,634	1,609	1,675	**Finnish**
763	859	657	630	15,486	11,195	1,041	1,016	**Mordovian**

Mordovian	Finnish	Estonian	Scottish	English	Orcadian	Norwegian	Icelandic
							0
						0	585
					0	1,115	928
				0	751	510	148
			0	688	1,303	1,302	823
		0	1,108	961	1,825	1,167	1,243
	0	899	1,689	1,571	2,221	1,765	1,623
0	1,678	708	1,537	1,340	1,903	1,611	1,451
